# Case Report: Graves' disease in a toddler with maple syrup urine disease: therapeutic challenges and surgical resolution

**DOI:** 10.3389/fped.2026.1803842

**Published:** 2026-05-11

**Authors:** Hebah Ghandora, Mays Khader Alzahrani, Rewa Lutfi Alsharif, Haifa Al Zahrani, Mohammed Al Garni, Hadeel Jambi

**Affiliations:** 1 College of Medicine, King Saud Bin Abdulaziz University for Health Sciences, Jeddah, Saudi Arabia; 2 King Abdullah International Medical Research Center, National Guard Health Affairs, Jeddah, Saudi Arabia; 3 Department of Pediatric Endocrinology, King Abdullah Specialized Children's Hospital, King Abdulaziz Medical City, National Guard Health Affairs, Jeddah, Saudi Arabia; 4 Department of Genetics and Precision Medicine, King Abdulaziz Medical City, National Guard Health Affairs, Jeddah, Saudi Arabia; 5 Department of ENT, King Abdulaziz Medical City, National Guard Health Affairs, Jeddah, Saudi Arabia; 6 Department of Clinical Nutrition, King Abdullah Specialized Children's Hospital, King Abdulaziz Medical City, National Guard Health Affairs, Jeddah, Saudi Arabia

**Keywords:** branched-chain amino acid (BCAA), glucose infusion rate (GIR), maple syrup urine disease (MSUD), thyroid function test (TFT), TRAb (Thyrotropin receptor antibodies)

## Abstract

A two-and-a-half-year-old girl with a known diagnosis of Maple Syrup Urine Disease (MSUD) developed challenging Graves' disease while undergoing liver transplant workup for definitive MSUD management. Initial low-dose methimazole failed to control hyperthyroidism; gradual dose escalation resulted in better control; however, the patient developed hepatotoxicity. Dose reduction caused poorly controlled hyperthyroidism, further complicated by severe neutropenia, necessitating discontinuation. In addition, multiple MSUD catabolic crises further complicated the medical management. A total thyroidectomy was planned after metabolic stabilization and preoperative preparation. Perioperative management included Lugol's iodine, hydrocortisone, and propranolol, but hyperthyroidism paradoxically worsened within 24 h due to the iodine effect. Neutrophil count improved with high-dose hydrocortisone; careful reintroduction of low-dose methimazole, with close monitoring of the ANC, resulted in better control of hyperthyroidism. Another perioperative challenge included strict branched-chain amino acid restriction and high-dose insulin infusion to counter hydrocortisone metabolic risk, with careful special “sick days” management guided by a metabolic specialist to prevent neurological sequelae and metabolic crisis. Total thyroidectomy proceeded uneventfully after multidisciplinary optimization. Postoperative transient hypocalcemia was managed conservatively, and levothyroxine replacement was initiated. The patient successfully underwent liver transplantation several months later.

## Case presentation

A two-and-a-half-year-old girl with a known diagnosis of Classical Maple Syrup Urine Disease (MSUD) was flagged by newborn screening and confirmed by whole exome sequencing (homozygous pathogenic splicing variant in the DBT gene, *NM_001918.2:c.773-2A>G*). She has been on MSUD dietary management since 4 days of age, with close follow-up and monitoring by the metabolic team. Infections triggered hospitalizations for metabolic decompensation, which standard MSUD sick-day management successfully managed. She was under evaluation for liver transplantation.

During one of the admissions for in-hospital sick day management in the context of viral illness, she was noticed to have unexplained tachycardia, with a heart rate ranging from 160 to 180 bpm. Her family history was notable for subclinical hypothyroidism in her father and hypothyroidism in her paternal grandmother. Thyroid function tests (TFTs) revealed hyperthyroidism (Table 1). Despite biochemical thyrotoxicosis, she exhibited none of the classic symptoms of hyperthyroidism, such as weight loss, hyperactivity, tremors, goitre, or ophthalmopathy. On physical examination, the thyroid gland was not palpable, and there was no tenderness or palpable cervical lymphadenopathy.

**Table 1:** Laboratory trends.

Parameter	Initial presentation	After initial management	On admission	Preoperative
	(At diagnosis)	(∼4 Months after diagnosis)	8 Months after diagnosis (at presentation with complications)	(Preoperative optimization)
TSH (0.5–5.0 mIU/L)	<0.01 mIU/L		<0.01 mIU/L	
Free T4 (12–22 pmol/L)	23.9 pmol/L	17.0 pmol/L	25.9 pmol/L	18.6 pmol/L
Free T3 (3.1–6.8 pmol/L)	6.8 pmol/L	6.2 pmol/L	29.9 pmol/L	4.1 pmol/L
ANC (1.5–8.0 × 10⁹/L)	7.67 × 10⁹/L	1.4 × 10⁹/L	0.41 × 10⁹/L	2.55 × 10⁹/L
AST	25 U/L	135 U/L	37 U/L	27 U/L
ALT (<40 U/L)	24 U/L	109 U/L	24 U/L	23 U/L

TSH, Thyroid-Stimulating Hormone; T4, Thyroxine; T3, Triiodothyronine; ANC, Absolute Neutrophil Count; AST, Aspartate Aminotransferase; ALT, Alanine Aminotransferase.

A thyroid ultrasound was unremarkable, but a radioactive iodine uptake scan showed nonuniform blood flow and multiple hypervascular foci in both lobes, consistent with a mildly hyperactive gland. Serologic testing confirmed Graves' disease, with a TSH receptor antibody (TRAb: 25 U/L, normal < 2 U/L). The diagnosis was further supported by a marked rise in TRAb levels at follow-up, reaching > 40 U/L at 6 months. The initially modest elevation in antibody levels and the absence of classical clinical features may be explained by the patient's young age and the early stage of disease at presentation.

Initial treatment included methimazole (0.25 mg/kg/day) and propranolol. Her condition was difficult to control, requiring close follow-up and frequent dose adjustments. She ultimately achieved euthyroidism on methimazole (1 mg/kg/day) four months after diagnosis. However, despite improved control of her hyperthyroidism, her metabolic control progressively worsened, with increasing off-target leucine levels despite optimized, age-appropriate caloric augmentation to meet the increased metabolic demands of thyrotoxicosis, as well as adequate protein and branched-chain amino acid (BCAA) intake with satisfactory adherence.

Nevertheless, the frequency of admissions for hyperleucinosis increased significantly, with some episodes complicated by thyrotoxic crises. She subsequently developed methimazole-induced transaminitis six weeks after a dose increase (Table 1). The methimazole dose was reduced to 0.8 mg/kg/day, and she was monitored closely. Her transaminase levels normalized; however, free T4 and free T3 levels began to rise, and she developed severe neutropenia eight months after diagnosis. Methimazole was therefore discontinued.

**Figure 1:**
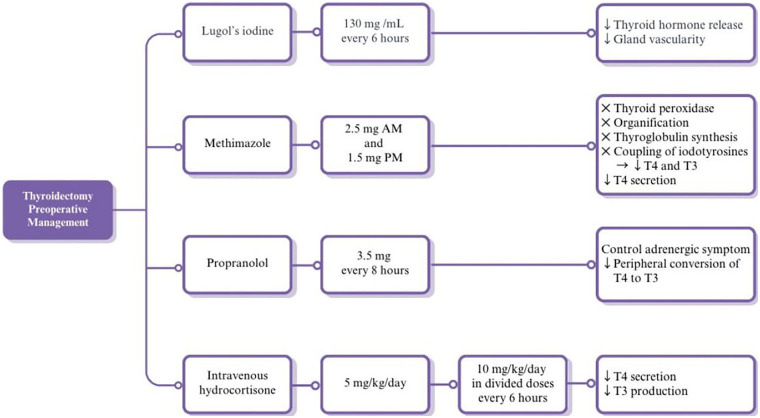
Preoperative management of hyperthyroidism. Schematic overview of the multimodal preoperative regimen used in a pediatric patient with Graves' disease. Treatment included Lugol's iodine to reduce thyroid hormone release and vascularity, methimazole to inhibit hormone synthesis, propranolol for symptomatic control and reduction of peripheral T4-to-T3 conversion, and intravenous hydrocortisone to decrease T3 production. Doses and mechanisms of action are illustrated, highlighting the strategy used to achieve rapid preoperative stabilization.

Due to the persistent biochemical hyperthyroidism, intolerance to oral antithyroid medications, and her overall unstable metabolic condition, alternative definitive management strategies were considered. In her case, radioactive iodine therapy was not favourable, given her young age (less than 5 years); thus, a total thyroidectomy emerged as the safest option. This approach aimed to optimize her thyroid status for better MSUD control—both are prerequisites for successful pre-liver transplantation preparation—while preventing complications, challenges, and comorbidities associated with uncontrolled hyperthyroidism and MSUD. She was electively admitted to the Pediatric Intensive Care Unit (PICU) for urgent preoperative optimization (Figure 1). On admission, she was stable, with a heart rate of 130 bpm and persistent hyperthyroidism (Table 1).

To reduce the risk of thyroid storm and prepare her for surgery, she was managed with Lugol's iodine, intravenous hydrocortisone, and propranolol, alongside close clinical and laboratory monitoring. Intravenous hydrocortisone was administered at 5 mg/kg/day, but because there was no response, the dose was slowly increased to 10 mg/kg/day, given in divided doses every 6 h. However, her thyroid hormone levels worsened, with free T4 increasing to 32.2 pmol/L and free T3 to 30.7 pmol/L. Her absolute neutrophil count improved to 1.4 × 10⁹/L following high-dose hydrocortisone therapy, allowing reintroduction of methimazole with close monitoring due to ongoing biochemical hyperthyroidism. Her free T4 and free T3 started to show improvement after methimazole reintroduction. Alongside high-dose hydrocortisone, high-dose Lugol's iodine, and beta blockers, she achieved a euthyroid state after 10 days. Lugol's iodine was discontinued after 10 days to avoid an “escape” and the re-synthesis of thyroid hormones.

From a metabolic perspective, upon admission, she was maintained on enteral feeds, a special milk formula, initially at 75% for the first 24 h of admission, then at 100% leucine with added isoleucine and valine supplements, after ensuring appropriate metabolic control with daily BCAAs levels. In addition, a side drip of intravenous fluids (IVF) was added to provide a total glucose infusion rate (GIR) of 10.7 mg/kg/min plus IV lipid emulsions of 20% (2–3 g/kg) over 24 h, providing a total caloric intake of 90 kcal/kg/day and a total protein intake of 2.7 g/kg/day. Insulin infusion 0.02–0.1 units/kg/h was added for anabolic effect during the clinically indicated IV hydrocortisone course while targeting blood glucose between 5 and 9 mmol/L. She was maintained on 50% leucine, supplemented with isoleucine and valine, and had her caloric intake augmented to 140 kcal/kg/d 24 h preoperatively. BCAAs were within treatment targets for plasma amino acid monitoring both preoperatively and up to 48 h postoperatively, as shown in the graph below.

On the day of surgery, her heart rate ranged between 95 and 120 bpm, and repeat labs showed an euthyroid state. During the preoperative NPO period, she was kept on D 12.5% IVF at one and a half maintenance rate, with GIR of 11 mg/kg/min with continued IV intralipids 3 g/kg over 24 h and titrated insulin infusion while maintaining her glucose levels within 5–9 mmol/L; this metabolic management was continued throughout the operative and postoperative phases until she was fit to resume enteral feeds. A stress dose of IV hydrocortisone (100 mg/m²/dose) was administered at induction of anesthesia. She underwent an uncomplicated total thyroidectomy under general anesthesia, and the procedure was completed without complications with minimal blood loss.

After the surgery, the patient remained hemodynamically stable and showed no signs of thyroid storm, bleeding, infection, or hoarseness. She developed transient hypocalcemia that required a single dose of IV calcium; she also received a short course of intravenous antibiotics (cefuroxime) and was initiated on levothyroxine replacement. Serial labs showed normal parathyroid hormone, corrected calcium, phosphorus, and magnesium, confirming the absence of post-total thyroidectomy complications such as hypoparathyroidism or hypocalcemia. She was given 50% leucine for the first 48 h after surgery. She maintained within-treatment targets for BCAAs levels through daily monitoring of plasma amino acids and successfully switched to 100% leucine intake on day 3 post-operation. Gradual weaning of intralipids, dextrose, IVF, and insulin was achieved, along with hydrocortisone. The surgical site healed well, and she was discharged in excellent condition after tolerating oral intake with a structured follow-up plan.

From the family's perspective, the clinical course was associated with significant anxiety due to treatment uncertainty and the risk of metabolic decompensation; however, they expressed reassurance with the multidisciplinary approach and overall satisfaction with the management and outcome.

On follow-up postoperatively, she was doing well. and her hypothyroidism was stable on levothyroxine 50 mcg daily. She underwent a successful liver transplant uneventfully, as a definitive management of MSUD, 5 months post-total thyroidectomy.

## Discussion

We describe the preoperative optimization of a pediatric patient with uncontrolled Graves' disease complicated by Maple Syrup Urine Disease (MSUD). The coexistence of these conditions poses unique therapeutic challenges, as standard pharmacologic strategies for controlling severe thyrotoxicosis may exacerbate metabolic instability in disorders of branched-chain amino acid metabolism.

For Graves' disease, antithyroid medications, specifically methimazole and propylthiouracil, remain the first-line treatments in both adult and pediatric populations (1, 2). Methimazole is generally preferred for its superior efficacy and lower incidence of side effects compared with propylthiouracil. Nevertheless, these medications carry the potential for rare but severe adverse effects, including agranulocytosis and hepatotoxicity, as well as more common minor reactions such as pruritus and urticaria (1, 2).

Neutropenia is characterized by an absolute neutrophil count (ANC) falling below 1.5 × 10⁹/L. Several factors may lead to neutropenia, including decreased production of neutrophils in the bone marrow, immune-mediated destruction of neutrophils, or pathological sequestration of neutrophils in tissues such as splenomegaly (3). Methimazole can also cause neutropenia by damaging bone marrow precursors, thereby stopping granulopoiesis and reducing neutrophil production. Additionally, immune-mediated destruction may occur via drug-dependent antibodies targeting neutrophils, leading to rapid reductions in ANC (4).

The timing of neutropenia is variable, and it is most commonly reported within 90 days after the initiation of methimazole (5). However, it may occur as early as six days or as late as ten years after starting the medication (6). Following discontinuation of methimazole, neutrophil counts typically recover within approximately 10 days (7).

Severe neutropenia is a rare but serious complication of antithyroid drugs (ATDs), and standard management requires immediate discontinuation of the offending drug without reintroduction due to the risk of recurrence and of life-threatening infections (8). Because of potential cross-reactivity among thioamides, switching to another ATD such as propylthiouracil or carbimazole is generally avoided (8). Although a few case reports describe cautious reintroduction of methimazole after improvement in absolute neutrophil count in highly selected situations—particularly in neonates and children with mild to moderate neutropenia and when alternative treatments are limited—large pediatric rechallenge studies are lacking, and the true recurrence risk remains unknown (9–11).

In our patient, alternative therapies were not tolerated, and thyroid function worsened without treatment, so complete discontinuation of methimazole was not feasible. Therefore, methimazole was carefully reintroduced at a very low dose with close hematologic monitoring. This approach underscores the therapeutic difficulties in pediatric patients when conventional treatment options are restricted, necessitating further research to establish optimal management strategies for ATD-associated neutropenia in children.

Current international guidelines recommend a multimodal pharmacologic approach for the management of severe thyrotoxicosis and thyroid storm, which is often extrapolated for rapid preoperative preparation in patients with Graves' disease. The European Thyroid Association describes four principal therapeutic components: an antithyroid drug, a *β*-adrenergic antagonist, inorganic iodine, and corticosteroids (12). Similarly, the American Thyroid Association recommends *β*-blockade—most commonly propranolol—because of its ability to control adrenergic symptoms and, at higher doses, inhibit peripheral conversion of thyroxine (T4) to triiodothyronine (T3) (2). The Japanese Thyroid Association also supports *β*-blocker therapy in severe thyrotoxicosis (1).

Inorganic iodine preparations are widely used in thyroid storm and short-term preoperative preparation for thyroidectomy, particularly when thionamides are contraindicated. High-dose iodine administered for 10–14 days suppresses thyroid hormone release through the Wolff–Chaikoff effect and reduces thyroid gland vascularity, thereby facilitating surgical management (13). However, an escape phenomenon commonly occurs after this period as adaptive mechanisms reduce active iodine transport into thyroid follicular cells, allowing thyroid hormone synthesis to resume once intrathyroidal iodine levels fall below the inhibitory threshold (13). Although higher iodine exposure has been proposed as a potential mechanism to overcome this escape through passive iodine transport into thyroid cells (14), paradoxical iodine-induced hyperthyroidism has been reported and was observed in our patient.

Glucocorticoids are another important component of therapy in severe thyrotoxicosis. Their effects include inhibition of peripheral conversion of T4 to the biologically active T3, support of adrenal function during severe metabolic stress, and prevention of relative adrenal insufficiency associated with hyperthyroidism (15, 16). Additional benefits may include improvement of vasomotor symptoms and potential support of hematologic recovery in cases of antithyroid drug–induced neutropenia (17). Adult guidelines commonly recommend hydrocortisone, typically administered as a 300 mg intravenous load, then 100 mg every 8 h (2). Japanese thyroid storm guidelines similarly recommend high-dose corticosteroid therapy in children, including dexamethasone at 0.6 mg/kg/d or hydrocortisone at 15 mg/kg/day in divided doses (1, 18). In pediatric practice, dosing is generally extrapolated from adult recommendations using weight-based regimens, commonly 5 mg/kg of hydrocortisone intravenously every 6–8 h (maximum 100 mg per dose) (16).

In our patient, treatment options were significantly limited because methimazole therapy had previously been complicated by hematologic adverse effects. Consequently, the antithyroid effect achievable with thionamides alone was limited, and additional pharmacologic strategies were required to achieve adequate biochemical control before surgery. Hydrocortisone was therefore initiated at 5 mg/kg/day and gradually increased to 10 mg/kg/day as an individualized strategy to enhance inhibition of peripheral thyroid hormone activation while providing perioperative stress-dose coverage.

In certain instances, adjunctive therapies may be considered. Bile acid sequestrants can decrease enterohepatic recycling of thyroid hormones, and plasmapheresis may be employed in refractory or life-threatening circumstances when standard pharmacologic therapy is ineffective (15, 19, 20). Despite these strategies, pediatric-specific guidance for rapid control of severe Graves' disease remains limited (21).

The coexistence of Maple Syrup Urine Disease (MSUD), an inherited disorder of branched-chain amino acid metabolism caused by a deficiency of the branched-chain *α*-ketoacid dehydrogenase complex, added significant complexity in the management of hyperthyroidism in this patient. During periods of physiologic stress and catabolic conditions, endogenous protein breakdown is stimulated, resulting in accumulation of the toxic branched-chain amino acids (leucine, isoleucine, and valine), predisposing patients with MSUD to metabolic decompensation and potential neurologic deterioration (22, 23). Severe thyrotoxicosis itself constitutes a hypercatabolic state characterized by increased energy expenditure, accelerated protein turnover, and enhanced proteolysis, all of which can aggravate the metabolic imbalance. In this context, the use of glucocorticoids—often indicated for thyroid storm or severe thyrotoxicosis—poses an additional challenge, given their potential to augment proteolysis and elevate circulating amino acid levels, thereby increasing the risk of metabolic instability in MSUD (24).

Although the identified variant is typically associated with intermediate or intermittent MSUD, our patient exhibited a phenotype consistent with classic disease, requiring dietary management from the neonatal period. This highlights the variability in genotype–phenotype correlation and is clinically relevant, as a more severe MSUD phenotype likely contributed to the significant metabolic instability observed during thyrotoxicosis, thereby increasing the complexity of co-management.

The use of glucocorticoids in this patient required careful balancing of their endocrine benefits against the risk of metabolic decompensation. To reduce this risk, insulin therapy was introduced as an anabolic support measure to limit endogenous protein catabolism. Insulin promotes amino acid uptake and protein synthesis, and inhibits proteolysis, thereby helping to counteract the catabolic effects of severe thyrotoxicosis and reduce branched-chain amino acid release in MSUD (25). This approach is consistent with metabolic management principles in MSUD and other organic acidemias, where caloric support and anabolic therapy are recommended during periods of physiologic stress (23, 26). In this patient, it allowed the beneficial effects of glucocorticoids while minimizing the risk of metabolic instability.

These factors highlight the delicate balance required in managing coexisting endocrine and metabolic disorders. Achieving adequate control of thyrotoxicosis while minimizing catabolic stress and treatment-related adverse effects necessitates close biochemical monitoring and a coordinated, multidisciplinary approach.

Incorporating the family's perspective was essential in guiding shared decision-making, particularly given the need to balance rapid biochemical control with the risk of precipitating metabolic decompensation. This was especially relevant when considering the reintroduction of methimazole and the use of glucocorticoids in a metabolically vulnerable patient.

## Conclusion

To our knowledge, this is the first reported pediatric case describing the coexistence of Graves' disease and MSUD requiring rapid preoperative stabilization in the setting of antithyroid drug intolerance. This case highlights the complex therapeutic challenge encountered and supports an individualized, multidisciplinary approach. It illustrates that appropriate clinical decisions balancing the risks and utilities of competing metabolic and endocrine priorities can be safely managed with careful biochemical monitoring and close coordination between endocrinology and metabolic teams, while emphasizing the need for pediatric-specific guidelines for rapid preoperative optimization in complex, high-risk endocrine scenarios.

## Data Availability

The original contributions presented in the study are included in the article/Supplementary Material, further inquiries can be directed to the corresponding author.
